# Supplementary material for: Slit-based irrigation catheters can reduce procedure-related ischemic stroke in atrial fibrillation patients undergoing radiofrequency catheter ablation
Source: PLoS One. 2020 Oct 1;15(10):e0239339. doi: 10.1371/journal.pone.0239339 (PMC7529237; doi:10.1371/journal.pone.0239339)
Supplement: S1 Fig — (PDF) [file pone.0239339.s001.pdf]

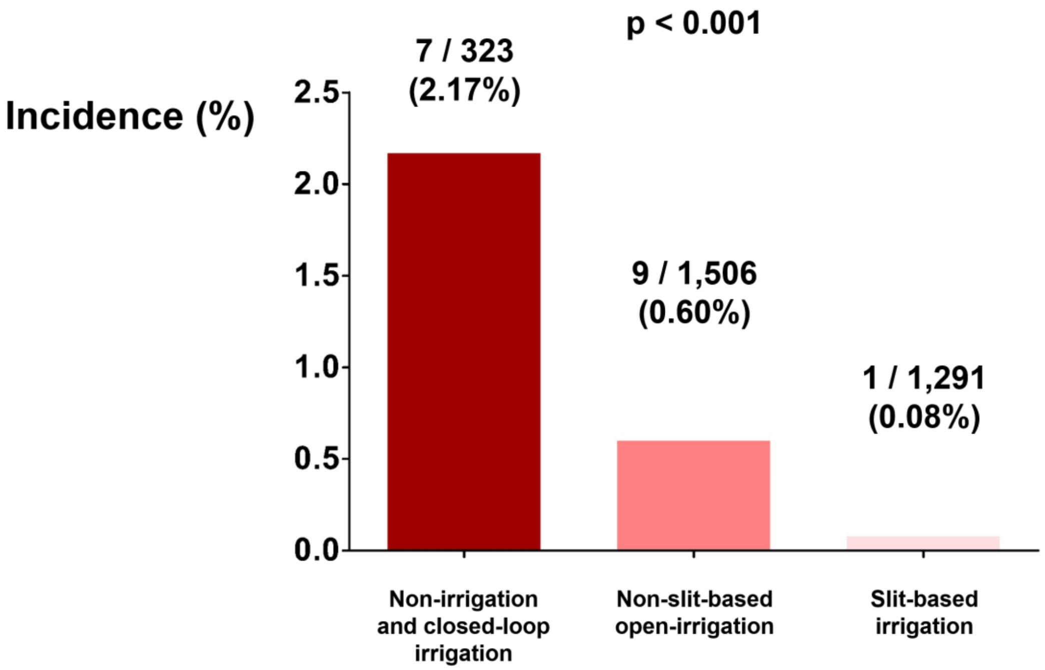

S1 Fig. Incidence of peri-procedural ischemic complication according to catheter type.

Closed-loop irrigation catheters were considered as non-irrigation catheter.
