# Supplementary material for: Slit-based irrigation catheters can reduce procedure-related ischemic stroke in atrial fibrillation patients undergoing radiofrequency catheter ablation
Source: PLoS One. 2020 Oct 1;15(10):e0239339. doi: 10.1371/journal.pone.0239339 (PMC7529237; doi:10.1371/journal.pone.0239339)
Supplement: S1 Table — (PDF) [file pone.0239339.s002.pdf]

S1 Table. Chronological difference among use of different catheter types.

	Time period			p value
	**Earlier third (1998.06 – 2010.10)**	**Mid third (2010.10 – 2015.03)**	**Later third (2015.03 – 2019.04)**	< 0.001
**Non-irrigation catheters**	288 (99.3%)	2 (0.7%)	0 (0%)	
**Non-slit-based irrigation catheters**	752 (48.9%)	484 (31.4%)	303 (19.7%)	
**Slit-based irrigation catheters**	0 (0%)	554 (42.9%)	737 (57.1%)	
