# Supplementary material for: Slit-based irrigation catheters can reduce procedure-related ischemic stroke in atrial fibrillation patients undergoing radiofrequency catheter ablation
Source: PLoS One. 2020 Oct 1;15(10):e0239339. doi: 10.1371/journal.pone.0239339 (PMC7529237; doi:10.1371/journal.pone.0239339)
Supplement: S2 Table — (PDF) [file pone.0239339.s003.pdf]

S2 Table. Multivariate model for procedure-related ischemic complication.

	Multivariate model (event n = 17)	
	**OR (95% CI)**	**p value**
**Slit-based irrigation catheter**	0.066 (0.008 – 0.528)	0.010
**Age (year)**	1.039 (0.987 – 1.095)	0.144
**Sex**	1.380 (0.384 – 4.962)	0.622
**Heart failure**	0.582 (0.074 – 4.578)	0.607
**Hypertension**	0.712 (0.248 – 2.042)	0.528
**Diabetes mellitus**	0.539 (0.070 – 4.169)	0.554
**Previous ischemic stroke/TIA**	1.438 (0.395 – 5.238)	0.582
**Vascular disease**	0.499 (0.062 – 4.001)	0.513
**Non-paroxysmal AF**	2.365 (0.688 – 8.131)	0.172
**Substrate modification**	0.828 (0.253 – 2.707)	0.755
**Pre-RFCA antithrombotics**	2.267 (0.884 – 5.811)	0.088
**Procedure period (earlier vs. mid vs. later third)**	0.807 (0.378 – 1.720)	0.578

Pre-RFCA antithrombotics were coded as follows: 1: no treatment; 2: antiplatelets; 3: warfarin; 4: NOAC.

Procedure periods were coded as follows: 1: earlier third; 2: mid third; 3: later third.

AF: atrial fibrillation; CI: confidence interval; NOAC: non-vitamin K oral anticoagulant; OR: odds ratio; RFCA: radiofrequency catheter ablation. Other abbreviations are the same as in the text.
