# Supplementary material for: Slit-based irrigation catheters can reduce procedure-related ischemic stroke in atrial fibrillation patients undergoing radiofrequency catheter ablation
Source: PLoS One. 2020 Oct 1;15(10):e0239339. doi: 10.1371/journal.pone.0239339 (PMC7529237; doi:10.1371/journal.pone.0239339)
Supplement: Attachment — Submitted filename: Revision Letter.docx [file pone.0239339.s004.docx]

Dear reviewers,

We appreciate your time and effort to provide insightful comments and suggestions that are kindly aimed to improve the quality of our manuscript. Your comments allowed us to view our own data from a different perspective and elaborate our analysis, which was an intellectually challenging experience. Point-by-point responses have been presented below each comment and included in the revised manuscript. Before you proceed to our responses to your comments, we would like to inform you that new contents included in the revised manuscript are highlighted by yellow background color for your convenience. We hope our responses would meet your requirements and expectation.

Sincerely yours,

Jaemin Shim, MD, PhD.

Dear editors of *PLoS One*,

We would like to thank you for taking the time and effort to review and process our manuscript. Insightful comments and suggestions made by the reviewer were well taken by all the authors. My colleagues and I have tried to address the point reviewer raised. Before you proceed to our responses to reviewer comments, we would like to inform you that new contents included in the revised manuscript are highlighted by yellow background color for your convenience. We believe that the changes we have made significantly improved the quality of our manuscript and we hope that the revised manuscript meets the standards of *PLoS One*.

Sincerely yours,

Jaemin Shim, MD, PhD.

Reviewer #1:

#1. The manuscript is quite interesting, BUT there is a big problem between what it is stated in the text and what one can read in table 3. For instance, in the text it is said that "slit - based" had "higher prevalence of SEC", but in table 3 incidence in slit based is 17% and in other is 21.5% (exatcly the opposite!).

- We appreciate your time and effort to review our manuscript.
- We apologize for our mistake. Slit-based catheter group had lower prevalence of SEC. We modified our text as appropriate which is as follows.
- Those who were ablated with slit-based irrigation catheter were older (56.65 ± 10.62 vs. 55.10 ± 11.15 years; p < 0.001); had larger LA (41.63 ± 6.18 vs. 40.89 ± 5.93 mm; p = 0.001); lower LAA flow velocity (46.59 ± 21.63 vs. 50.08 ± 21.15 cm/sec; p < 0.001); and lower prevalence of spontaneous echo-contrast (17.0% vs. 21.5%; p = 0.002) and vascular disease (5.0% vs. 9.6%; p < 0.001); and higher prevalence of non-paroxysmal AF (45.5% vs. 37.7%; p < 0.001). **[page 14, line 3 – 9]**
- We included SEC in our multivariate model. The results are as follows.

**Table 4.** Multivariate model for procedure-related ischemic complication.

|  | **Model 1 (event n = 15)** | | **Model 2 (event n = 17)** | |
| --- | --- | --- | --- | --- |
|  | **OR (95% CI)** | **p value** | **OR (95% CI)** | **p value** |
| **Slit-based irrigation catheter** | 0.073 (0.009 – 0.562) | 0.012 | 0.070 (0.009 – 0.536) | 0.010 |
| **Age (year)** | 1.019 (0.962 – 1.079) | 0.525 | 1.041 (0.989 – 1.097) | 0.123 |
| **Sex** | 1.158 (0.306 – 4.380) | 0.829 | 1.398 (0.389 – 5.032) | 0.608 |
| **Heart failure** | 0.535 (0.064 – 4.482) | 0.564 | 0.642 (0.083 – 4.977) | 0.672 |
| **Hypertension** | 0.653 (0.208 – 2.056) | 0.467 | 0.737 (0.258 – 2.108) | 0.570 |
| **Diabetes mellitus** | 0.634 (0.081 – 4.980) | 0.665 | 0.532 (0.069 – 4.109) | 0.545 |
| **Previous ischemic stroke/TIA** | 1.732 (0.467 – 6.418) | 0.411 | 1.504 (0.412 – 5.484) | 0.536 |
| **Vascular disease** | 0.562 (0.071 – 4.466) | 0.586 | 0.472 (0.060 – 3.685) | 0.474 |
| **Non-paroxysmal AF** | 2.011 (0.462 – 8.747) | 0.352 | 2.388 (0.678 – 8.409) | 0.175 |
| **Substrate modification** | 0.732 (0.190 – 2.829) | 0.651 | 0.796 (0.245 – 2.587) | 0.704 |
| **Left atrial diameter (mm)** | 1.042 (0.945 – 1.149) | 0.410 | Not included | Not included |
| **Flow velocity of Left atrial appendage (cm/sec)** | 0.992 (0.960 – 1.025) | 0.624 | Not included | Not included |
| **SEC** | 1.385 (0.425 – 4.514) | 0.589 | Not included | Not included |
| **Pre-RFCA anticoagulation** | 5.151 (0.623 – 42.606) | 0.128 | 2.375 (0.612 – 9.213) | 0.211 |

OR: odds ratio. Other abbreviations are the same as in the text.

#2. Another important issue is that "other catheters" were less anticoagulated BEFORE ablation (55.6% vs 67.3%) and remained less anticoagulated AFTER ablation (93.5% vs 97.2%) and both values were statistically significant.

- This is another important issue. As you mentioned, there were difference in anticoagulation status between slit vs. non-slit catheters. However, in our cohort anticoagulation status was not a significant determinant of procedure-related ischemic stroke which is presented in Figure 2. Multivariate analysis model also suggested that slit-based irrigation catheter was associated with reduced risk of procedure-related ischemic stroke despite difference in baseline characteristics including pre-RFCA anticoagulation status.

#3. Finally, incidence of vascular diseases was almost twice as high in "other catheters" group. This point should be highlighted in the discussion Thus, or there is some mistake in the table or this could represent an important bias in judging superiority of the slit-based catheters. Authors must explain this discrepancy.

- We appreciate your important suggestions. Since our cohort is a non-randomized registry based, there can be differences in baseline demographics and unmeasured confounders. We added this issue in the limitation section.
- Although the prevalence of vascular disease was significantly different between the two groups, overall CHA_2_DS_2_-VASc score was well matched. Although the prevalence of SEC was in favor of slit-based group, LA diameter, AF type, LAA flow velocity, was in favor of non-slit-based catheter group. Patients with vascular disease had numerically lower risk of procedure-related stroke (0.4% vs. 0.6%; OR = 0.749; p = 0.780).
- Differences in baseline characteristics such as prevalence of vascular disease, AF type, LA diameter, and LAA flow velocity were observed between slit-based vs. non-slit based catheter groups although it was adjusted in the multivariate model. Unmeasured confounders might exist in our cohort. **[page 19, line 18 – 21]**

Reviewer #2: Kim et al present the results of a retrospective study of peri-procedural ischemic stroke in 3120 patients undergoing first RF catheter ablation at a single center over 20 years. They conclude that use of slit-based irrigated catheters was associated with a lower incidence of ischemic stroke, and this was independent of other risk factors in multivariate analysis.

Questions and comments:
#1. How do the authors account for the possibility of changes in operative technique over the 20+ year period of analysis? If non-irrigated catheters were used predominantly in the early years, followed by non-slit-based irrigated catheters, followed by irrigated catheters, there might have been other differences that were not accounted for but correlated with the type of irrigation. For example, was the ACT target identical from 1998 to 2009? Was the timing of the heparin bolus the same, or did operators wait until after transseptal access was achieved in the early years? Was perioperative oral anticoagulation completely constant over this long time period? Were any changes made to sheath aspiration/flush technique, or number/type of diagnostic catheters? How many ablation catheters incorporated contact-force sensing? Was there a difference in warfarin vs NOAC use?

- We appreciate your time and effort to review our manuscript.
- We understand your concern. As you mentioned, ablation techniques and anticoagulation protocols have improved over time. However, the basics of the procedure such as trans-septal puncture, heparin bolus / infusion during the procedure, and sheath flushing techniques did not change. Our strategy was to give bolus heparin (100 IU per kg) before trans-septal access even in 1990s. Target ACT was constantly 300 – 350 sec. Several institutions now use continuous flushing of SL1 or deflectable sheath during the procedure. However, we do not use continuous sheath flushing technique. This strategy did not change over time in our institution. Minor variation can exist in the number of diagnostic catheters based on the discretion of the operator. However, no diagnostic catheters were placed in the left atrium. Only ablation and mapping catheters were placed in the left atrium.
- The major change over the study period is the introduction of NOAC. Anticoagulation with warfarin had significant limitations such as under- or over-anticoagulation. Anticoagulation has become a more reliable and constant after use of NOAC. However, we would like to state that pre- and post-RFCA anticoagulation strategy did not affect the occurrence of procedure-related stroke as depicted in Figure 2. This is probably due to performance of TEE in majority of the patients (92.9%). Although there are variations in anticoagulation strategy over time, routine evaluation with TEE could have attenuated the influence of variations in anticoagulation strategy.

#2. Why do authors lump together internally irrigated catheters and external non-slit-based irrigation catheters? These are very different designs, in fact more dissimilar than slit-based and non-slit-based irrigation catheters, and it seems unwarranted to include them in a single group for analysis.

- This is another insightful comment. A total of 33 patients underwent RFCA with closed-loop irrigation catheters. As recommended, we performed additional analysis with closed-loop irrigation included in the non-irrigation group. The results are presented as Supplementary Figure S1. The results were similar with slit-based irrigation group having the lowest risk for procedure-related stroke.
- The results were identical when closed-loop irrigation catheters were classified as non-irrigation catheters (S1 Fig.). **[page 15, line 13 – 14]**

**Supplementary Fig. S1.** Incidence of peri-procedural ischemic complication according to catheter type.

Closed-loop irrigation catheters were considered as non-irrigation catheter.

#3. How many of the 17 ischemic complications occurred >24 hours after the ablation procedure?

- In our cohort, a total of 11 patients had procedure-related stroke > 24 hours after RFCA. 6 patients had procedure-related stroke within 24 hours after RFCA. We added the time to first event in Table 2. Thirteen patients had procedure-related stroke within 3 days.

#4. What proportion of patients in the non-irrigated, non-slit-based irrigated and slit-based irrigated groups had substrate ablation in addition to PVI? Why was substrate ablation not included as a variable in the multivariate model presented in Table 4?

- We are pleased to have these important discussions. The percentage of patients underwent substrate modification was similar between slit-based group vs. non-slit based group as described in Table 3. The percentage substrate modification among non-irrigated, non-slit-based irrigated, and slit-based irrigated groups are 25.6%, 49.8%, and 44.9%, respectively.
- We modified our Table 4. We included SEC and substrate modification in the model. The modified table is also included in this revision letter in the issue #5.

#5. The text says the slit-based irrigated group had higher prevalence of SEC, but Table 3 says it was lower. Which is correct? Why was SEC not included as a variable in the multivariate model presented in Table 4?

- This issue was also raised by the reviewer 1. We corrected our mistake.
- Slit-based catheter group had lower prevalence of SEC. We modified our text as appropriate which is as follows. We also included SEC in our multivariate model (Table 4) which is also shown below.
- Those who were ablated with slit-based irrigation catheter were older (56.65 ± 10.62 vs. 55.10 ± 11.15 years; p < 0.001); had larger LA (41.63 ± 6.18 vs. 40.89 ± 5.93 mm; p = 0.001); lower LAA flow velocity (46.59 ± 21.63 vs. 50.08 ± 21.15 cm/sec; p < 0.001); and lower prevalence of spontaneous echo-contrast (17.0% vs. 21.5%; p = 0.002) and vascular disease (5.0% vs. 9.6%; p < 0.001); and higher prevalence of non-paroxysmal AF (45.5% vs. 37.7%; p < 0.001). **[page 14, line 3 – 9]**

**Table 4.** Multivariate model for procedure-related ischemic complication.

|  | **Model 1 (event n = 15)** | | **Model 2 (event n = 17)** | |
| --- | --- | --- | --- | --- |
|  | **OR (95% CI)** | **p value** | **OR (95% CI)** | **p value** |
| **Slit-based irrigation catheter** | 0.073 (0.009 – 0.562) | 0.012 | 0.070 (0.009 – 0.536) | 0.010 |
| **Age (year)** | 1.019 (0.962 – 1.079) | 0.525 | 1.041 (0.989 – 1.097) | 0.123 |
| **Sex** | 1.158 (0.306 – 4.380) | 0.829 | 1.398 (0.389 – 5.032) | 0.608 |
| **Heart failure** | 0.535 (0.064 – 4.482) | 0.564 | 0.642 (0.083 – 4.977) | 0.672 |
| **Hypertension** | 0.653 (0.208 – 2.056) | 0.467 | 0.737 (0.258 – 2.108) | 0.570 |
| **Diabetes mellitus** | 0.634 (0.081 – 4.980) | 0.665 | 0.532 (0.069 – 4.109) | 0.545 |
| **Previous ischemic stroke/TIA** | 1.732 (0.467 – 6.418) | 0.411 | 1.504 (0.412 – 5.484) | 0.536 |
| **Vascular disease** | 0.562 (0.071 – 4.466) | 0.586 | 0.472 (0.060 – 3.685) | 0.474 |
| **Non-paroxysmal AF** | 2.011 (0.462 – 8.747) | 0.352 | 2.388 (0.678 – 8.409) | 0.175 |
| **Substrate modification** | 0.732 (0.190 – 2.829) | 0.651 | 0.796 (0.245 – 2.587) | 0.704 |
| **Left atrial diameter (mm)** | 1.042 (0.945 – 1.149) | 0.410 | Not included | Not included |
| **Flow velocity of Left atrial appendage (cm/sec)** | 0.992 (0.960 – 1.025) | 0.624 | Not included | Not included |
| **SEC** | 1.385 (0.425 – 4.514) | 0.589 | Not included | Not included |
| **Pre-RFCA anticoagulation** | 5.151 (0.623 – 42.606) | 0.128 | 2.375 (0.612 – 9.213) | 0.211 |

OR: odds ratio. Other abbreviations are the same as in the text.

#6. Since irrigated catheters are no longer used for AF ablation, and the slit-based irrigated catheters analyzed here (CoolFlex and Flexability) do not incorporate contact-force sensing, the Discussion should include some comment on the trade-off of giving up contact-force sensing for slit-based irrigation, including new lesion metrics (Ablation Index, Lesion Size Index) that rely on these catheters.

- We appreciate your kind comments. As you mentioned, slit-based catheters do not have contact-force sensing capability. Therefore, Ablation Index (CARTO) and Lesion Size Index (Abbott) parameters are also unavailable. We added these discussions in our manuscript.
- Slit-based irrigation catheters do not have contact-force sensing capability. Therefore, ablation parameters such as ablation index or lesion size index which has shown to improve ablation outcome cannot be utilized when using slit-based irrigation catheters [21,22]. This can be a critical limitation of slit-based irrigation catheters and contact-force sensing ability should be added in the near future. **[page 20, line 3 – 7]**

Reviewer #3: The authors use a retrospective analysis of a single center large database to provide data that slit-like irrigation is associated with reduced rates of thromboembolic complications following AF ablation compared to other irrigated and non-irrigated catheters. The overall number of patients undergoing ablation is large, although this database spans 20 years of practice with a number of systematic changes over time. Stroke or TIA occurred significantly less with irrigated catheters than non-irrigated catheters. More importantly, slit-like irrigation appeared to be associated with less events compared to non-slit irrigation catheters despite similar number of cases. If true, this is an important observation.

#1. The major limitation is the contemporaneous changes in clinical practice, especially anticoagulation during the 20 years of analysis. As practice evolves, often a whole-system change occurs at a center making one catheter and non-fluoroscopic mapping system dominant for a particular time. This can make an untended impact on outcomes in a retrospective analysis like this. Specifically, the introduction and use of uninterrupted anticoagulation during this period. Specifically, the number of patients undergoing ablation with uninterrupted anticoagulation needs to be very similar between groups of irrigated catheters for any confidence that there is a real difference in the irrigation architecture as a mechanism of the difference in outcome. If this cannot be assured, it becomes very difficult to know if the difference should drive changes in practice.

- We appreciate your time and effort to review our manuscript.
- We understand your concern. As you mentioned, ablation techniques and anticoagulation protocols have improved over time. However, the basics of the procedure such as trans-septal puncture, heparin bolus / infusion during the procedure, and sheath flushing techniques did not change. Our strategy was to give bolus heparin (100 IU per kg) before trans-septal access even in 1990s. Target ACT was constantly 300 – 350 sec. Several institutions now use continuous flushing of SL1 or deflectable sheath during the procedure. However, we do not use continuous sheath flushing technique. This strategy did not change over time in our institution. Minor variation can exist in the number of diagnostic catheters based on the discretion of the operator. However, no diagnostic catheters were placed in the left atrium. Only ablation and mapping catheters were placed in the left atrium.
- The major change over the study period is the introduction of NOAC. Anticoagulation with warfarin had significant limitations such as under- or over-anticoagulation. Anticoagulation has become a more reliable and constant after use of NOAC. However, we would like to state that pre- and post-RFCA anticoagulation strategy did not affect the occurrence of procedure-related stroke as depicted in Figure 2. This is probably due to performance of TEE in majority of the patients (92.9%). Although there are variations in anticoagulation strategy over time, routine evaluation with TEE could have attenuated the influence of variations in anticoagulation strategy.
- The percentage of patients having pre-RFCA anticoagulation were different between slit-based irrigation vs. other catheters as described in Table 3. However, we would like to emphasize that this difference was adjusted in the multivariate model (Table 4) and pre-RFCA anticoagulation did not affect procedure-related stroke (Figure 2). In contrast to previous reports pre-RFCA anticoagulation was not an important factor in terms of preventing procedure-related stroke in our cohort. This is probably due to high performance rate of TEE.

#2. The number of outcomes are very small. As such, a small difference in confounding practice (like OAC use during the procedure) can have a major impact. As such, the authors need to make it clear if the catheter types, especially the different irrigated catheters, were used at the same time or overlapping time periods. More importantly, a clear analysis of the number of patients undergoing uninterrupted OAC is required.

- We understand your concern. Event number in our cohort is limited. However, procedure-related stroke rate after RFCA is low and it should be low. Furthermore, there will be no randomized clinical trials comparing different catheters in terms of procedure-related stroke mainly due to low event rate which requires significant large number of enrollment. This type of retrospective analysis is virtually the only way to compare irrigation efficacy of different type of catheters.
- There was chronological overlap between catheter types. Since slit-based irrigation catheters were introduced most recently they were mostly used in the later period. These results are summarized as follows.
- Although there was significant chronological difference among catheter types, this was not an independent determinant of procedure-related stroke in the multivariate model as shown below (Supplementary Table S2).

**Supplementary Table S1.** Chronological difference among use of different catheter types.

|  | **Time period** | | | **p value** |  |
| --- | --- | --- | --- | --- | --- |
|  | **Earlier third**  **(1998.06 – 2010.10)** | **Mid third**  **(2010.10 – 2015.03)** | **Later third**  **(2015.03 – 2019.04)** | < 0.001 | |
| **Non-irrigation catheters** | 288  (99.3%) | 2  (0.7%) | 0  (0%) |  | |
| **Non-slit-based irrigation catheters** | 752  (48.9%) | 484  (31.4%) | 303  (19.7%) |  | |
| **Slit-based irrigation catheters** | 0  (0%) | 554  (42.9%) | 737  (57.1%) |  | |

- Antithrombotic treatment in our cohort was either no therapy, antiplatelets, warfarin, or NOAC. We included this variable as four components in the multivariate model instead of just with or without anticoagulation. The results were similar and included as Supplementary Table S2.
- The influence of chronological variations in the use of different type of ablation catheters were also adjusted and type of ablation catheter remained the only independent variable for procedure-related ischemic complication (Supplementary Table S1 and S2). **[page 15, line 16 – 18]**

**Supplementary Table S2.** Multivariate model for procedure-related ischemic complication.

|  | **Multivariate model (event n = 17)** | |
| --- | --- | --- |
|  | **OR (95% CI)** | **p value** |
| **Slit-based irrigation catheter** | 0.066 (0.008 – 0.528) | 0.010 |
| **Age (year)** | 1.039 (0.987 – 1.095) | 0.144 |
| **Sex** | 1.380 (0.384 – 4.962) | 0.622 |
| **Heart failure** | 0.582 (0.074 – 4.578) | 0.607 |
| **Hypertension** | 0.712 (0.248 – 2.042) | 0.528 |
| **Diabetes mellitus** | 0.539 (0.070 – 4.169) | 0.554 |
| **Previous ischemic stroke/TIA** | 1.438 (0.395 – 5.238) | 0.582 |
| **Vascular disease** | 0.499 (0.062 – 4.001) | 0.513 |
| **Non-paroxysmal AF** | 2.365 (0.688 – 8.131) | 0.172 |
| **Substrate modification** | 0.828 (0.253 – 2.707) | 0.755 |
| **Pre-RFCA antithrombotics** | 2.267 (0.884 – 5.811) | 0.088 |
| **Procedure period**  **(earlier vs. mid vs. later third)** | 0.807 (0.378 – 1.720) | 0.578 |

Pre-RFCA antithrombotics were coded as follows: 1: no treatment; 2: antiplatelets; 3: warfarin; 4: NOAC.

Procedure periods were coded as follows: 1: earlier third; 2: mid third; 3: later third.

OR: odds ratio. Other abbreviations are the same as in the text.

#3. It is recognized that the slit irrigation group had worse risk factors that would confound by making the thromboembolic rates higher in this group. It might also make the authors more likely to use uninterrupted OAC for this group.

- This is another important comment. As you mentioned, overall anticoagulation rate was higher in slit-based irrigation group. However, we would like to state that these difference in anticoagulation strategy was adjusted in the multivariate model (Table 4).
- More importantly, antithrombotic strategy before RFCA did not influence the occurrence of procedure-related stroke as depicted in Figure 2. As we mentioned earlier, this might be due to performance of TEE in the majority of patients (92.9%).

#4. There are other limitations in this kind of analysis that cannot be clarified including possible systematic differences in the ascertainment of stroke over time in a database.

- This is another important issue. Shortcomings originating from retrospective cohort analysis is the limitation of our study.
- The diagnosis criteria and tools such as diffusion weighted imaging has been established for decades and did not change over time during our study period. Residents and fellows of cardiology and neurology changes annually. This might have influence on the ascertainment of stroke over time in our database. However, the correctness of ascertainment of stroke or TIA was reviewed during data collection. All brain MRI or CT imaging taken during admission and emergency department visit was also reviewed. We consider systematic differences in the ascertainment of stroke over time in our cohort is not significant. However, this important issue is added in the limitation section.
- Systematic variations in the ascertainment of stroke or TIA over time might exist since our cohort extends more than 20 years. However, all stroke or TIA events were diagnosed by neurologists and this was centrally reviewed during data collection process. **[page 19 – 20, line 21 – 1]**

#5. What specific definition of stroke was used as opposed to TIA and mimickers like migraine? Ordinarily TIA is not used as a hard endpoint, although there is only one TIA among the outcomes.

- We appreciate your important comments. The major differentiation between stroke and TIA was the presence of acute infarction lesion in brain imaging. The final diagnosis was made by neurologist.
- As you mentioned, a lot of symptom mimicking stroke or TIA happens after RFCA such as headache, migraine, or dizziness. Therefore, acute lesion in brain imaging is critical for the diagnosis of procedure-related complication.
- Patient 15 in our cohort was diagnosis of with TIA. This patient has definite sudden weakness in left arm. We immediately consulted our neurologist and acute stroke was highly suspected. However, brain MRI revealed nothing and his symptoms was fortunately improved. This patient was diagnosed with TIA by our neurologist since initial symptom had highly localizing feature.

#6. Coolflex catheters do not have force measuring capabilities. As such, authors have traded off this function for different irrigation. This raises the importance of the veracity of this data as a change to a slit irrigation ablation eliminates an ablation technology important at many centers.

- We understand your concern. Slit-based irrigation catheter lacks contact-force sensing capability. Probably due to technical limitation, Abbott added contact-force sensing capability to 6-hole irrigation system. It may be harder to incorporate contact-force sensing system into slit-based irrigation tip since the tip itself bends (which is in fact a strong point in terms of efficient irrigation). Fortunately, slit-based irrigation system with contact-force sensing capability will be available in near future under the name of TactiFlex. We added these discussions in the limitation section.
- Slit-based irrigation catheters do not have contact-force sensing capability. Therefore, ablation parameters such as ablation index or lesion size index which has shown to improve ablation outcome cannot be utilized when using slit-based irrigation catheters [21,22]. This can be a critical limitation of slit-based irrigation catheters and contact-force sensing ability should be added in the near future. **[page 20, line 3 – 7]**
